# Prevalence and sociodemographic predictors of high-risk vaginal human papillomavirus infection: findings from a public cervical cancer screening registry

**DOI:** 10.1186/s12889-023-17132-2

**Published:** 2023-11-14

**Authors:** Anis-Syakira Jailani, Nur Zahirah Balqis-Ali, Kar Foong Tang, Weng Hong Fun, Shazimah Abdul Samad, Rohaidza Jahaya, Nurun Najihah Subakir, Roziah Ismail, Zakiah Mohd Said, Sondi Sararaks

**Affiliations:** 1 Centre for Health Outcomes Research, Institute for Health Systems Research, National Institutes of Health, Ministry of Health Malaysia, Block B2, No. 1, Jalan Setia Murni U13/52, Seksyen U13 Bandar Setia Alam, Shah Alam, Selangor 40170 Malaysia; 2 Family Health Section, Family Health Development Division, Ministry of Health, Malaysia, Putrajaya, 62590 Malaysia

**Keywords:** Human papillomavirus (HPV), HPV Infection, Cervical cancer, Prevalence, Malaysia, Community-based screening

## Abstract

**Introduction:**

High-risk human papillomavirus (HPV) screening is vital for early cervical cancer detection and treatment. With the introduction of the national cervical cancer screening programme and screening registry in Malaysia, there is a need to monitor population-based HPV screening uptake and high-risk HPV prevalence as part of cervical cancer surveillance.

**Objective:**

To determine the prevalence and sociodemographic factors predicting high-risk HPV infection in Malaysia based on a public, community-based cervical cancer screening registry targeting women at risk of getting HPV infection.

**Methods:**

The study used data from the Malaysian cervical cancer screening registry established by the Family Health Development Division from 2019 to 2021. The registry recorded sociodemographic data, HPV test details and results of eligible women who underwent HPV screening at public primary healthcare facilities. A vaginal sample (via self-sampling or assisted by a healthcare provider) was used for DNA extraction for HPV detection and genotyping. Registry data were extracted and analysed to determine prevalence estimates of high-risk HPV infection. Multifactorial logistic regression analysis was conducted to determine predictors of high-risk HPV infection. All analyses were performed using Stata version 14.

**Results:**

The programme screened a total of 36,738 women during the study period. Women who attended the screening programme were mainly from urban areas, aged 30–39 years, and of Malay ethnicity. The prevalence of high-risk HPV infection was 4.53% among women screened, with the yearly prevalence ranging from 4.27 to 4.80%. A higher prevalence was observed among urban settling women, those aged 30–49 years, those of Indian ethnicity, and those without children. The results from logistic regression showed that women from urban areas, lower age groups, of Indian or Chinese ethnicity, and who are self-employed were more likely to be infected with high-risk HPV.

**Conclusion:**

Targeted and robust strategies to reach identified high-risk groups are needed in Malaysia. In addition, the registry has the potential to be expanded for an improved cervical cancer elimination plan.

**Trial registration:**

Trial registration number: NMRR ID-22-00187-DJU.

**Supplementary Information:**

The online version contains supplementary material available at 10.1186/s12889-023-17132-2.

## Introduction

Cervical cancer remains one of the most common cancers and a leading cause of mortality among women globally, with most cases and deaths occurring in low- and middle-income countries [1–3]. Persistent infection with high-risk human papillomavirus (HPV) is the primary etiologic factor for cervical cancer, with HPV types 16 and 18 accounting for more than 70% of all cervical cancers [4, 5]. Given the association between high-risk HPV infection and cervical cancer, HPV screening has become one of the key targets in the World Health Organization’s (WHO) strategy for cervical cancer elimination [6].

In Malaysia, cervical cancer is the third most common cancer and the fourth leading cause of cancer mortality among women, with an age-standardised incidence rate of 10.2 per 100,000 women (1,740 new cases) and an age-standardised mortality rate of 5.8 per 100,000 women (991 deaths) in 2020 [7]. There was a decrease in the 5-year cumulative cervical cancer incidence from 2012 to 2016 (3,981 cases) compared with 2007 to 2011 (4,352 cases) [8]. Despite the decreasing trend, around 40% of cases were detected at late stages (stages III and IV), with the peak incidence occurring among women aged 50 to 65 [8]. This highlights that improving cervical cancer screening in Malaysia is paramount to ensure earlier detection and treatment.

The approach towards cervical cancer screening in Malaysia was based on Papanicolaou (Pap) Smear testing since 1969 [9]. However, the screening rate remains low, hovering around 16–25%  (KKM: 100-24/4/4 /(21) MESYUARAT SUSULAN FOCUS GROUP 2 : STRENGTHENING HEALTHCARE DELIVERY SERVICES BAGI KAJIAN SEPARUH PENGGAL (KSP) RANCANGAN MALAYSIA KE-12 (KSP) RMKe-12) BAB 4 UNTUK KESIHATAN, unpublished) due to the opportunistic nature of the programme, patient and health system-related barriers, and the unavailability of a national screening registry to document screening [10]. In response to low screening rates and the need for scale-up of screening for cervical cancer elimination, the Ministry of Health (MOH) Malaysia has adopted the WHO recommendation of transitioning to HPV-based testing, which has been shown to have higher sensitivity, cost-effectiveness, and better feasibility through self-sampling and longer interval between screenings due to increased negative predictive value for high-grade cervical intra-epithelial neoplasia compared with Pap Smear [11–14]. HPV testing will replace Pap Smear as the primary screening method by 2023 in public healthcare facilities under MOH Malaysia, as outlined in MOH Malaysia’s cervical cancer elimination plan to achieve the target of 70% screening coverage of women using a high-performance test [6, 9].

With the shift in cervical cancer screening towards more practical testing, free HPV DNA-based testing has been made available at healthcare facilities under MOH Malaysia through the national cervical cancer screening programme [9, 15]. The programme was initiated in four states in 2019 and has since been expanded to 12 out of 13 states and three federal territories in Malaysia in 2022. This move preceded the establishment of a national cervical cancer screening registry by the Family Health Development Division, MOH Malaysia, in 2019 to document screening initiatives within public primary healthcare facilities and improve cervical cancer screening programme monitoring in Malaysia [13, 16].

HPV screening and prevalence reporting are essential for identifying high-risk subgroups in population-based cervical cancer surveillance for health policy and decision-making [6, 17]. Within the literature, the prevalence of high-risk HPV infection was reported to be 5% globally [18], with similar prevalence rates reported in Southeast Asian countries, such as Singapore, Thailand, and Vietnam [18–21]. The prevalence of high-risk HPV infection in Malaysia was reported to be between 4 and 11% [22–24], with studies differing in sample size, study setting, HPV genotypes reported, and test method. Under the dichotomous Malaysian healthcare system, a challenge exists in determining high-risk HPV prevalence. Data on screening conducted in private healthcare facilities need to be integrated or captured in the national screening registry [25]. Nevertheless, with the majority of screening tests conducted in the public healthcare system and the introduction of the national cervical cancer screening programme and registry, determining prevalence based on the national screening registry would provide an updated high-risk HPV prevalence estimate at the population level.

Thus, this study aimed to determine the prevalence and sociodemographic factors predicting high-risk HPV infection in Malaysia based on a public, community-based cervical cancer screening registry. To our knowledge, this is the first attempt to determine the prevalence of high-risk HPV infection through a national screening registry in Malaysia. The findings will provide insight for policymakers in streamlining the national cervical cancer elimination action plan [9] and improving Malaysia’s cervical cancer screening programme. It would contribute to a better understanding of HPV infection prevalence as we report findings from a Southeast Asian country with a multi-ethnic population that may differ from other settings.

## Method

### Study design and sampling population

This cross-sectional study analysed data from the Malaysian cervical cancer screening registry by the Family Health Development Division, MOH Malaysia. The registry recorded data from public primary healthcare facilities from selected states in Malaysia that have been conducting HPV testing as part of the national cervical cancer screening programme since its introduction in 2019 [10, 16].

### Data collection and registry reporting

Trained healthcare professionals at public primary healthcare facilities meticulously recorded screening information in a standardised Excel spreadsheet. This information encompassed comprehensive details about women undergoing screening and their corresponding results. The collected data were compiled and submitted to the Family Health Development Division, MOH, which examined, verified, and validated the information. The sociodemographic and economic factors captured in the registry and used in this study were age, nationality, ethnicity, highest education level, occupation, household income, and the number of children. However, locality, education level, occupation, and household income data were only collected from August 2020 and designated as ‘unrecorded values’ to signify the absence of prior data. The name of the healthcare facility and state were recorded, and the location status (urban or rural) was generated based on the address of the healthcare facility. Other data include the date and method of sample collection and the date of results. The data analysed for this study were up until December 2021. They included six states (Wilayah Persekutuan Kuala Lumpur & Putrajaya, Selangor, Negeri Sembilan, Kelantan, Kedah, and Johor) implementing HPV testing between 2019 and 2021. A full description of data collected by the registry is in Additional file 1.

### Screening approach

Women attending public primary healthcare facilities identified as at risk of developing HPV infection were approached to conduct HPV testing. The screening guide was as follows: i) women aged between 30–65 years, ii) of Malaysian nationality, iii) had sexual intercourse experience, iv) have an intact uterus and v) never had cervical cancer screening or the last screening was more than three years ago. Despite the age criteria, some states screened women between 20–29 years old who fulfilled all other criteria; they were included in this study for comprehensiveness. The women visited either the outpatient or maternal-child health department at the public primary healthcare facility for a regular follow-up of health condition, postnatal follow-up or walk-in for any disease or condition. All eligible women were approached and briefed about HPV testing by an in-house healthcare provider. Consent was obtained before testing.

### Vaginal sample collection

Women were encouraged to perform the test themselves via self-sampling or assisted by the healthcare provider. In the self-sampling procedure, the healthcare provider briefed women on the sampling procedure using a schematic diagram that came with the kit. Two types of testing kits were employed: a dry Flocked Swab model 552C/80mm (Copan Italia S.p.a) for samples destined for analysis in government-owned laboratories and a Viba-brush (Rovers Medical Devices) for samples to be outsourced to private laboratories. Recruited women were provided with one of the testing kits. They would need to sample by removing the swab from the tube, inserting it into the vagina, making a few rotational brushing movements, and placing the swab back into the tube [15]. The collected sample would then be handed over to the healthcare provider. All samples were sent to public or private laboratories for HPV DNA analysis and notification of results.

### HPV DNA detection and genotyping

The samples were tested through the Roche Cobas 4800 HPV test (Cobas) at the laboratories. This novel molecular method utilises real-time PCR (RT-PCR) through a fully automated system, allowing the detection of 14 high-risk HPV genotypes, including HPV16, HPV18, and 12 other high-risk HPVs (HPV31, -33, -35, -39, -45, -51, -52, -56, -58, -59, -66, and − 68) reported as a pooled result and β-globin as the control for extraction and amplification adequacy. The results were reported as ‘positive’ for high-risk HPV (HPV 16, HPV 18 or other non-16/18 high-risk HPV types) or ‘negative’ for high-risk HPV or ‘unsatisfactory’. ‘Unsatisfactory’ resulted from incorrect labelling, missing form, insufficient material for testing, inhibition by blood or other substance, or damaged specimen. The test has shown good sensitivity, accuracy, and reproducibility [26, 27]. It fulfils all requirements set by international guidelines and is considered a validated method for cervical cancer screening [27].

### Results and subsequent actions

An appointment was set to inform the results. HPV testing results were reported as either unsatisfactory HPV test, high-risk HPV not detected, high-risk HPV positive 16/18 type detected, or other high-risk non-16/18 HPV types detected. In the case of an unsatisfactory sample, women were requested to repeat the test. Multiple infections were not recorded, whereby if a woman were found to be infected by multiple HPV genotypes, she would be assigned to only one group: high-risk 16/18 or other high-risk non-16/18 HPV. When HPV 16/18 and other high-risk types were positive, the result was assigned as HPV 16/18 positive. If a woman is detected as high-risk non-16/18 HPV positive, she will be scheduled for a follow-up cytology test at the same facility. The woman would then be advised on the next step, including a referral to a specialist in the case of an abnormal cytology result. On the other hand, if a woman is detected to be high-risk HPV 16/18 positive, she will be referred for colposcopy at the nearest hospital [15].

### Data analysis

The characteristics of women who attended and performed HPV testing were first described to gain insight into the population who attended public primary healthcare facilities and participated in the screening programme. The characteristics were compared to the population distribution data reported by the Department of Statistics Malaysia (DOSM) [28] to highlight the differences in characteristics of those who accessed public healthcare services from the general population. Prevalence estimates of high-risk HPV infection were reported for (1) overall prevalence, (2) prevalence based on the year of data collection, (3) prevalence based on each sociodemographic and economic factor, and (4) prevalence based on different types of HPV infection (HPV 16/18 and other non-16/18 high-risk types). The characteristics and prevalence were compared with previous studies conducted in Malaysia to understand the impact of the study setting, different timelines, and other factors on prevalence findings. All responses were included, with missing values recorded in the descriptive analysis.

The factors were then entered into a multifactorial logistic regression analysis to determine the potential predictors of high-risk HPV infection. Missing data (2.9%, *n* = 1,067) were dropped from the logistic regression analysis. Before conducting multifactorial logistic regression, simple logistic regressions were performed between each independent variable and high-risk HPV infection. Crude odd ratios (OR) were used to estimate the strength of the association (Additional file 2). The final multifactorial logistic regression included variables with a *p*-value < 0.25 in the simple logistic regression [29]. In the final model, the enter variable selection method was employed to identify predictors due to the constraints imposed by the limited number of factors available. Reference groups were selected based on ease of interpretation of the results or groups with the most significant samples. A two-tailed *p*-value of < 0.05 was regarded as statistically significant. Multicollinearity was checked using the variance inflation factor (VIF), with an acceptable value set at 10 [30]. The model’s predictive ability was assessed using the area under the curve (AUC) of the receiver operating characteristic curves (ROC), with a value of more than 0.6 being acceptable accuracy [31]. The model goodness of fit was tested using Hosmer–Lemeshow statistics, and a *p*-value > 0.05 was considered a good fit [32]. All analyses were performed in Stata version 14 (Stata Corp, College Station, TX, USA).

## Results

The programme screened a total of 36,738 women during the study period. The characteristics of women who attended the screening are shown in Table 1. Among the six states that implemented HPV testing by the end of 2021, Wilayah Persekutuan Kuala Lumpur and Putrajaya, the capital state of Malaysia, had the highest attendance (48.20%). The majority were from urban localities (63.28%), aged between 30–39 years (58.46%), of Malay ethnicity (83.40%), and had three or fewer children (a total of 70.50%). A high percentage of unrecorded values were observed for locality, education level, occupation, and household income, as these data were not collected in the registry during the initial stage (before August 2020). However, the recorded data showed that many attendees were from lower-income groups and were either government employees or not working. The distribution by state, locality, and ethnicity differed from the population distribution reported by DOSM, highlighting that the characteristics of those attending the screening programme differ from those of the general population. Approximately 48.20% of attendees were from Wilayah Persekutuan Kuala Lumpur and Putrajaya, as opposed to 11.23% of the total population residing there. In comparison, 77.69% of Malaysians reside in urban areas, as opposed to 86.95% of women attending the screening in the study. Only 8.29% of the study participants were Chinese, while the population distribution was 22.40%.

**Table 1:** Profile and distribution of women attending cervical cancer screening via HPV *DNA* testing between 2019–2021

Variables	n	%	% By population distribution^¶^
**Total**			
Overall	36,738	100.00	
**Locality**^†^			
Rural	3,489	9.50 (13.01) ^a^	22.31
Urban	23,248	63.28 (86.95) ^a^	77.69
Unrecorded^#^	10,001	27.22	
**State**			
Johor	979	2.66	21.62
Kedah	9,392	25.56	12.13
Kelantan	2,410	6.56	10.37
Negeri Sembilan	1,252	3.41	6.66
Selangor	4,997	13.60	37.98
WPKL & Putrajaya	17,708	48.20	11.23
**Age group (years)**			
20–29	587	1.60	28.15
30–39	21,478	58.46	28.19
40–49	11,073	30.14	21.02
50–65	3,600	9.80	22.64
**Ethnicity**			
Malay	30,638	83.40	69.80
Chinese	3,046	8.29	22.40
Indian	2,447	6.66	6.80
Others	591	1.61	1.00
Missing	16	0.04	
**Education level**^†^			
Never attended school/Primary	655	1.78 (5.75) ^a^	9.50
Secondary	4,816	13.11 (42.27) ^a^	50.00
Certificate/Tertiary	5,923	16.12 (51.98) ^a^	40.50
Unrecorded^#^	25,344	68.99	
**Income level**^†^			
< = RM3999	6,656	18.12 (58.47) ^a^	Monthly household income in the year 2020: Median = RM5209; Mean = RM7089
RM4000-RM7999	3,637	9.90 (31.95) ^a^	
> = RM8000	1,091	2.97 (9.58) ^a^	
Unrecorded^#^	25,354	69.01	
**Occupation**^†^			
Self-employed	758	2.06 (6.65) ^a^	-
Government employee	3,675	10.00 (32.26) ^a^	
Private employee	2,665	7.25 (23.39) ^a^	
Pensioner/Housewife	4,294	11.69 (37.69) ^a^	
Unrecorded^#^	25,346	68.99	
**Sampling method**			
Assisted by HCP	826	2.25	-
Self-sampling	35,872	97.64	
Missing	40	0.11	
**Year of HPV screening**			
2019	17,493	47.62	-
2020	9,073	24.70	
2021	10,172	27.69	
**Number of children**			
0	2,444	6.70	-
1	4,813	13.10	
2	8,820	24.00	
3	9,816	26.70	
4	6,246	17.00	
> 4	4,380	11.90	
Missing	219	0.60	
**HPV results**			
High-risk HPV not detected	34,005	92.56	-
Positive high-risk HPV 16/18	452	1.23	
Positive high-risk non-16/18 HPV	1,214	3.30	
Total high-risk HPV positive	1,666	4.53	
Unsatisfactory HPV test	911	2.48	
Missing	156	0.42	

*HCP* Healthcare provider, *RM* Malaysian Ringgit, *WPKL* Wilayah Persekutuan Kuala Lumpur, *DOSM* Department of Statistics Malaysia, *n* number, *%* percentage

^¶^ The percentage of women attending the screening was compared to population data for 2020 to determine the difference in characteristics of those attending the public screening program from the general population

^†^ Information on locality, education level, income level and occupation was only collected from August 2020 onwards, rendering many unrecorded cases marked as ^#^

^a^ Percentage after removing unrecorded cases

The overall and yearly prevalence are shown in Table 2. The total prevalence of high-risk HPV infection, regardless of the high-risk genotypes, between 2019 and 2021 among the sampled population was 4.53%. The yearly prevalence did not differ much, with fewer women screened in 2020 due to the COVID-19 pandemic.

**Table 2:** Prevalence of high-risk HPV infection according to the year of screening

Year of HPV screening	Total, n	High-risk HPV positive			High-risk HPV not detected	Unsatisfactory test	Missing
		Total	16/18	Non-16/18			
		n (%)	n (%)	n (%)	n (%)	n (%)	n (%)
2019	17,493	791 (4.52)	229 (1.31)	562 (3.21)	16,205 (92.64)	451 (2.58)	46 (0.26)
2020	9,073	387 (4.27)	97 (1.07)	290 (3.20)	8,299 (91.47)	336 (3.70)	51 (0.56)
2021	10,172	488 (4.80)	126 (1.24)	362 (3.56)	9,501 (93.4)	124 (1.22)	59 (0.58)
Overall	36,738	1,666 (4.53)	452 (1.23)	1,214 (3.30)	34,005 (92.56)	911 (2.48)	156 (0.42)

*n* number, *%* percentage

**Fig. 1:**
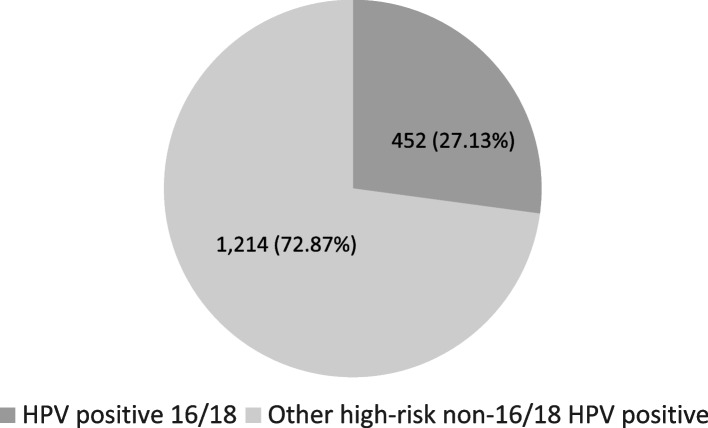
Proportion of HPV16/18 and other high-risk non-16/18 HPV among women found to have a high-risk HPV infection

The prevalence of high-risk HPV infection based on sociodemographic and economic factors is reported in Table 3. The prevalence was higher among urban settlers (5.57%) than rural settlers (2.52%). Two states were found to have a high prevalence: Johor (7.56%) and Wilayah Persekutuan Kuala Lumpur and Putrajaya (6.07%). Variations were seen across major Malaysian ethnicities, with Indians having the highest prevalence (8.58%), followed by Chinese (7.32%) and Malay (3.86%). Self-employed people had a higher prevalence (6.73%) than other job categories. We found the prevalence to decrease with an increasing number of children, with the highest prevalence among women without children (9.21%). We further described the characteristics by categorising HPV infection by HPV16/18 and other high-risk non-16/18 HPV types (Additional file 3). The overall prevalence was 1.23% for HPV16/18 and 3.30% for other high-risk non-16/18 types among those screened. This reflects 27.13% of HPV 16/18 among women detected with high-risk HPV infection (Fig. 1).

**Table 3:** Prevalence of high-risk HPV infection based on the socio-demographic and economic profile of women attending screening (*n* = 36,738)

Variables	Total, n	High-risk HPV not detected		High-risk HPV Positive		Unsatisfactory HPV test		Missing		*P*-value ^a^
		**n**	**% (95% CI)**	**n**	**% (95% CI)**	**n**	**% (95% CI)**	**n**	**% (95% CI)**	
**Total**										
Overall	36,738	34,005	92.56 (92.29–92.82)	1,666	4.53 (4.33–4.75)	911	2.48 (2.33–2.64)	156	0.42 (0.36–0.50)	
**Locality**^†^										
Rural	3,489	3,295	94.44 (93.63–95.15)	88	2.52 (2.05–3.10)	84	2.41 (1.95–2.97)	22	0.63 (0.42–0.96)	0.000*
Urban	23,248	21,307	91.65 (91.29–92.00)	1,296	5.57 (5.29–5.88)	562	2.42 (2.23–2.62)	83	0.36 (0.29–0.44)	
Unrecorded^#^	10,001	9,403	94.02 (93.54–94.47)	282	2.82 (2.51–3.16)	265	2.65 (2.35–2.98)	51	0.51 (0.39–0.67)	
**State**										
Johor	979	854	87.23 (84.99–89.18)	74	7.56 (6.06–9.39)	26	2.66 (1.81–3.87)	25	2.55 (1.73–3.75)	0.000*
Kedah	9,392	8,829	94.01 (93.51–94.47)	266	2.83 (2.52–3.19)	248	2.64 (2.33–2.98)	49	0.52 (0.39–0.69)	
Kelantan	2,410	2,275	94.40 (93.41–95.25)	50	2.07 (1.58–2.73)	77	3.20 (2.56–3.98)	8	0.33 (0.17–0.66)	
Negeri Sembilan	1,252	1,149	91.77 (90.12–93.17)	55	4.39 (3.39–5.68)	39	3.12 (2.28–4.24)	9	0.72 (0.37–1.38)	
Selangor	4,997	4,794	95.94 (95.35–96.45)	147	2.94 (2.51–3.45)	39	0.78 (0.57–1.07)	17	0.34 (0.21–0.55)	
WPKL & Putrajaya	17,708	16,104	90.94 (90.51–91.36)	1,074	6.07 (5.72–6.43)	482	2.72 (2.49–2.97)	48	0.27 (0.20–0.36)	
**Age group (years)**										
20–29	587	529	90.12 (87.43–92.29)	25	4.26 (2.89–6.23)	22	3.75 (2.48–5.63)	11	1.87 (1.04–3.35)	0.088
30–39	21,478	19,901	92.66 (92.30–93.00)	996	4.64 (4.36–4.93)	490	2.28 (2.09–2.49)	91	0.42 (0.35–0.52)	
40–49	11,073	10,194	92.06 (91.54–92.55)	511	4.61 (4.24–5.02)	323	2.92 (2.62–3.25)	45	0.41 (0.30–0.54)	
50–65	3,600	3381	93.92 (93.09–94.65)	134	3.72 (3.15–4.39)	76	2.11 (1.69–2.64)	9	0.25 (0.13–0.48)	
**Ethnicity**										
Malay	30,638	28,564	93.23 (92.94–93.51)	1182	3.86 (3.65–4.08)	759	2.48 (2.31–2.66)	133	0.43 (0.37–0.51)	0.000*
Chinese	3,046	2,764	90.74 (89.66–91.72)	223	7.32 (6.45–8.30)	47	1.54 (1.16–2.05)	12	0.39 (0.22–0.69)	
Indian	2,447	2,145	87.66 (86.29–88.90)	210	8.58 (7.53–9.76)	84	3.43 (2.78–4.23)	8	0.33 (0.16–0.65)	
Others	591	519	87.82 (84.92–90.22)	50	8.46 (6.47–10.99)	19	3.21 (2.06–4.99)	3	0.51 (0.16–1.56)	
Missing	16	13	81.25 (54.23–94.06)	1	6.25 (0.82–35.04)	2	12.50 (3.00–39.76)	0		
**Education level**^†^										
Never attended school/Primary	655	607	92.67 (90.41–94.44)	37	5.65 (4.12–7.70)	9	1.37 (0.72–2.62)	2	0.31 (0.08–1.21)	0.084
Secondary	4,816	4,467	92.75 (91.99–93.45)	252	5.23 (4.64–5.90)	75	1.56 (1.24–1.95)	22	0.46 (0.30–0.69)	
Certificate/Tertiary	5,923	5,526	93.30 (92.63–93.91)	260	4.39 (3.90–4.94)	93	1.57 (1.28–1.92)	44	0.74 (0.55–1.00)	
Unrecorded^#^	25,344	23,405	92.35 (92.02–92.67)	1,117	4.41 (4.16–4.67)	734	2.90 (2.70–3.11)	88	0.35 (0.28–0.43)	
**Occupation**^†^										
Self-employed	758	690	91.03 (88.77–92.87)	51	6.73 (5.15–8.75)	11	1.45 (0.81–2.60)	6	0.79 (0.36–1.75)	0.002*
Government employee	3,675	3,433	93.41 (92.57–94.17)	152	4.14 (3.54–4.83)	71	1.93 (1.53–2.43)	19	0.52 (0.33–0.81)	
Private employee	2,665	2,461	92.35 (91.27–93.30)	151	5.67 (4.85–6.61)	33	1.24 (0.88–1.74)	20	0.75 (0.48–1.16)	
Pensioner/Housewife	4,294	4,014	93.48 (92.70–94.18)	195	4.54 (3.96–5.21)	62	1.44 (1.13–1.85)	23	0.54 (0.36–0.80)	
Unrecorded^#^	25,346	23,407	92.35 (92.02–92.67)	1,117	4.41 (4.16–4.67)	734	2.90 (2.70–3.11)	88	0.35 (0.28–0.43)	
**Income level**^†^										
< = RM3999	6,656	6,189	92.98 (92.34–93.57)	327	4.91 (4.42–5.46)	95	1.43 (1.17–1.74)	45	0.68 (0.51–0.9)	0.443
RM4000-RM7999	3,637	3,375	92.80 (91.91–93.59)	177	4.87 (4.21–5.62)	69	1.90 (1.50–2.40)	16	0.44 (0.27–0.72)	
> = RM8000	1,091	1,027	94.13 (92.57–95.38)	44	4.03 (3.01–5.38)	13	1.19 (0.69–2.04)	7	0.64 (0.31–1.34)	
Unrecorded^#^	25,354	23,414	92.35 (92.01–92.67)	1,118	4.41 (4.16–4.67)	734	2.90 (2.70–3.11)	88	0.35 (0.28–0.43)	
**Sampling method**										
Assisted by HCP	826	755	91.40 (89.29–93.13)	41	4.96 (3.67–6.67)	23	2.78 (1.86–4.16)	7	0.85 (0.40–1.77)	0.515
Self-sampling	35,872	33,215	92.59 (92.32–92.86)	1,623	4.52 (4.31–4.74)	885	2.47 (2.31–2.63)	149	0.42 (0.35–0.49)	
Missing	40	35	87.50 (73.04–94.76)	2	5.00 (1.23–18.18)	3	7.50 (2.40–21.07)	0		
**Year of HPV screening**										
2019	17,493	16,205	92.64 (92.24–93.01)	791	4.52 (4.22–4.84)	451	2.58 (2.35–2.82)	46	0.26 (0.2–0.35)	0.337
2020	9,073	8,299	91.47 (90.88–92.03)	387	4.27 (3.87–4.70)	336	3.70 (3.33–4.11)	51	0.56 (0.43–0.74)	
2021	10,172	9,501	93.40 (92.90–93.87)	488	4.80 (4.40–5.23)	124	1.22 (1.02–1.45)	59	0.58 (0.45–0.75)	
**Number of children**										
0	2,444	2,128	87.07 (85.68–88.34)	225	9.21 (8.12–10.42)	82	3.36 (2.71–4.15)	9	0.37 (0.19–0.71)	0.000*
1	4,813	4,407	91.56 (90.75–92.32)	261	5.42 (4.82–6.10)	119	2.47 (2.07–2.95)	26	0.54 (0.37–0.79)	
2	8,820	8,176	92.70 (92.14–93.22)	382	4.33 (3.93–4.78)	220	2.49 (2.19–2.84)	42	0.48 (0.35–0.64)	
3	9,816	9,142	93.13 (92.62–93.62)	417	4.25 (3.87–4.67)	213	2.17 (1.90–2.48)	44	0.45 (0.33–0.60)	
4	6,246	5,855	93.74 (93.11–94.31)	215	3.44 (3.02–3.92)	150	2.40 (2.05–2.81)	26	0.42 (0.28–0.61)	
> 4	4,380	4,095	93.49 (92.72–94.19)	159	3.63 (3.12–4.23)	118	2.69 (2.25–3.22)	8	0.18 (0.09–0.36)	
Missing	219	202	92.24 (87.86–95.13)	7	3.20 (1.53–6.56)	9	4.11 (2.15–7.72)	1	0.46 (0.06–3.18)	

*HCP* Healthcare provider, *RM* Malaysian Ringgit, *WPKL* Wilayah Persekutuan Kuala Lumpur, *n* number, *%* percentage, *95% CI* 95% confidence interval

^a^ Chi-square test compared high-risk HPV positive detected versus high-risk HPV not detected groups

^†^ Information on locality, education level, income level and occupation was only collected from August 2020 onwards, rendering many unrecorded cases marked as ^#^

^*^ *p*-value < 0.05

We describe the differences in HPV prevalence among a few studies conducted in Malaysia in Table 4. We found that the high-risk HPV infection prevalence was similar to that in the hospital-based study by Othman et al. [23] but lower than that in the other two studies [22, 24]. The setting, HPV genotypes tested, sample size and targeted women who were screened differed across studies, with the current study reporting the most significant number of women screened. The prevalence was highest in the youngest age group (less than 25 or 30 years) in the other studies, while in the current study, the prevalence was highest in the age group 30 to 39 years.

**Table 4:** Comparison of HPV infection prevalence across different studies conducted in Malaysia

	Current study	Khoo et al. [22]	Othman et al. [23]	Rahmat et al. [24]
Screening programme	Community-based	Community-based	Hospital-based	Laboratory-based
Screening method	HPV DNA testing	HPV DNA testing	Pap Smear	HPV DNA testing
HPV genotypes tested	14 High-risk (HPV 16, 18, 31, 33, 35, 39, 45, 51, 52, 56, 58, 59, 66, and 68)	14 High-risk (HPV 16, 18, 31, 33, 35, 39, 45, 51, 52, 56, 58, 59, 66, 68) and 2 low-risk (6, 11)	14 High-risk (HPV 16, 18, 31, 33, 35, 39, 45, 51, 52, 56, 58, 59, 66, and 68), 6 low-risk (6, 11, 40, 43, 44, 54,70), and undefined HPV risk	23 High-risk (16, 18, 26, 31, 33, 35, 39, 45, 51, 52, 53, 55, 54, 56, 58, 59, 66, 68, 69, 70, 72, 73, 82), 10 low-risk types: (6, 11, 40, 42, 43, 44, 61, 62,81, 84), and 1 undefined
Target population	Women attending outpatient or maternal-child health departments of public primary healthcare facilities from six states in Malaysia	Women attending outpatient departments at five public primary healthcare facilities in Selangor.	Women attending three main hospitals in the North-Eastern region of West Malaysia	HPV DNA results were retrieved from archives in the Lab Information System (LIS) of Gnosis Laboratory from six states in Malaysia
Sector	Public	Public	Public	Private
Number of women screened	36,738	1,256	635	764
Duration	3 years (2019–2021)	3 years (2013–2015)	Not mentioned	2 years (2017–2018)
The targeted age group for screening (years)	20–65	18–60	Not mentioned Mean ± SD age among women screened: 43 ± 10.5	20–74
Prevalence	4.53% (high-risk genotypes)	7.20% (all tested genotypes) 6.50% (high-risk genotypes)	4.40% (all tested genotypes) 3.77% (high-risk genotypes)	14.00% (all tested genotypes) 10.70% (high-risk genotypes)
Prevalence by age group	20–29: 4.26% 30–39: 4.64% 40–49: 4.61% 50–65: 3.72%	18–24: 11.84% 25–30: 6.28% 31–40: 8.02% 41–50: 5.74% 51–60: 7.32%	Not described	21–29: 15.90% 30–65: 13.90% >65: 0

*SD* Standard deviation

The results from logistic regression are reported in Table 5. Locality, age group, ethnicity, and occupation were significant predictors of high-risk HPV infection. Urban settlers had a 1.48 times higher likelihood (adjusted odds ratio (aOR): 1.48, 95% confidence interval (CI): 1.09 to 2.00, *p* = 0.011) of having high-risk HPV infection than those from rural areas. All younger age groups were significantly more likely to be infected when compared to those aged 50 to 65, with those aged 20 to 29 having the highest likelihood (aOR: 2.57, 95% CI: 1.48 to 4.46, *p* = 0.001). Compared to Malays, Indians and Chinese were more likely to be infected (aOR):1.92, 95% CI: 1.49 to 2.48, *p* < 0.001 and aOR: 1.67, 95% CI: 1.30 to 2.13, *p* < 0.001, respectively). Only those who were self-employed were found to have significantly higher odds of being infected when compared to government employees (aOR: 1.57, 95% CI: 1.09 to 2.28, *p* = 0.016). States, education level and household income had no significant association with the risk of infection.

The number of children was dropped from the model due to significant association with age, tested through a chi-square test (*X*^2^ = 884.91, df = 15, *p*-value < 0.001). Assessment of the logistic regression found the AUC to be 0.66, indicating sufficient accuracy of the model in predicting the outcome [32]. The Hosmer-Lemeshow goodness-of-fit test was found to be not significant (*p* = 0.965) specifying a good model fit. The mean variance inflation factor (VIF) stood at 1.21, and all VIF values for the independent variables were below 10, ranging from 1.07 to 1.47 (Additional file 4). These results strongly indicate that multicollinearity is highly unlikely.

**Table 5:** Factors associated with high-risk HPV infection among women attending the screening programme (*n* = 11,047)

Variables	Multivariable logistic regression		
		**Adjusted OR (95% CI)**	***P*-value**
**Locality**			
Rural	ref		
Urban		1.48 (1.09–2.00)	0.011*
**State**			
Kelantan	ref		
Johor		1.72 (0.89–3.30)	0.104
Kedah		omitted^a^	
Negeri Sembilan		1.11 (0.58–2.14)	0.748
Selangor		0.54 (0.29–1.02)	0.058
WPKL & Putrajaya		1.31 (0.69–2.48)	0.415
**Age group (years)**			
20–29		2.57 (1.48–4.46)	0.001*
30–39		1.60 (1.24–2.08)	0.000*
40–49		1.85 (1.42–2.42)	0.000*
50–65	ref		
**Ethnicity**			
Malay	ref		
Chinese		1.67 (1.30–2.13)	0.000*
Indian		1.92 (1.49–2.48)	0.000*
Others		1.61 (0.98–2.66)	0.062
**Education level**			
Never attended school/Primary		1.36 (0.90–2.04)	0.142
Secondary		1.17 (0.94–1.46)	0.151
Certificate/Tertiary	ref		
**Occupation**			
Government employee	ref		
Self-employed		1.57 (1.09–2.28)	0.016*
Private employee		1.27 (0.98–1.66)	0.074
Pensioner/Housewife		1.09 (0.82–1.43)	0.557
**Income level**			
< = RM3999		1.06 (0.74–1.52)	0.739
RM4000-RM7999		1.18 (0.84–1.67)	0.343
> = RM8000	ref		
**Pseudo r**^**2**^			
0.04			
**Mean VIF**			
1.21			
**AUC**			
0.66			

*OR* Odds ratio, *95% CI* 95% confidence interval, *RM* Malaysian Ringgit, *WPKL* Wilayah Persekutuan Kuala Lumpur, *VIF* Variance inflation factor, *AUC* Area under the curve

^a^ Multicollinearity with locality

^*^ *p*-value < 0.05

## Discussion

We analysed a public, community-based, high-risk HPV screening registry. Those who attended the HPV screening programme were mainly from urban areas, aged between 30–39 years old, and of Malay ethnicity. The prevalence of high-risk HPV infection was 4.53% among women screened, with the yearly prevalence ranging from 4.27 to 4.80% between 2019 and 2021. The prevalence was highest among urban settling women, in Johor and Wilayah Persekutuan Kuala Lumpur and Putrajaya states, those aged between 30–49 years, of Indian ethnicity, those self-employed, and those without children. Those from urban areas, lower age groups, of Indian or Chinese ethnicity, and self-employed were more likely to be infected with high-risk HPV.

Most women who attended the screening were urban settlers, of Malay ethnicity and aged between 30 and 39 years. The distribution by state, locality, and ethnicity of women who attended the programme differed from the population distribution. In the dichotomous health systems of Malaysia, where public and private health sectors operate independently, this finding represents women who tend to attend screening at public primary healthcare facilities. This finding corroborated the characteristics of outpatient users described by a local study, highlighting that 68.60% of women used public healthcare facilities. In comparison, the remaining 31.40% used private facilities and, thus, are unlikely to be reached by the programme [33]. The same study highlighted that non-Malay ethnicities use private healthcare facilities more frequently, which should be considered because the prevalence of high-risk HPV found in this study was higher in non-Malay ethnicities. Given that adherence to cervical cancer screening was similar across all ethnicities [34], the prevalence finding for non-Malays, especially Chinese, may be inaccurate if many had the screening done in the private sector. Therefore, it is desirable that this registry also captures private sector data. However, due to the variability of notification and reporting by the private sector, the accuracy and completeness of data remain a challenge. The findings support the design and need for a more targeted yet effective screening programme to reach high-risk groups.

In this study, the estimated prevalence of 4.53% was slightly higher than the prevalence reported by a hospital-based study conducted in Malaysia in 2014 by Othman et al. (3.77%) [23] but lower when compared with two other studies reporting 6.50% (Khoo et al.) and 10.70% (Rahmat et al.) [22, 24]. Both the current study and Khoo et al. tested 14 high-risk HPV genotypes. Rahmat et al. tested 23 high-risk genotypes, possibly explaining the higher prevalence. However, the 14 high-risk genotypes included in the current study were the most common genotypes known to cause more than 80% of all HPV infections [35]. Instead, the large sample size, community-based setting, and inclusion of more study sites and geographical locations in the registry may better estimate the prevalence. Prevalence estimates based on cross-sectional studies are limited by various factors, including heterogeneity of the sample [36], with a study reporting the HPV prevalence to vary as much as 20 times among different regions [37], thus necessitating an extensive and comprehensive sample as used in the current study. Nonetheless, the study by Rahmat et al. was the only one conducted in the private sector, which may influence the estimated prevalence, thus warranting future exploration involving both sectors. The different time points in each study may further affect the prevalence. However, our study showed slight variation across several years. It is worth noting that a few other Malaysian studies have reported on HPV prevalence, specifically among individuals with abnormal pathology [38, 39]. These studies were not directly compared with the present one, as the current study primarily centres on the prevalence among generally healthy women participating in a screening programme.

The estimated prevalence is comparable and slightly lower than the global prevalence rate. The overall adjusted prevalence of high-risk HPV infection was 5.00% based on a meta-analysis involving 1,016,719 women globally [18]. It is also comparable to three neighbouring countries of Malaysia, with a high-risk HPV infection prevalence of 5.05% in Singapore [20], 5.40% in Thailand [19], and 5.00% in Myanmar [40]. There was significant heterogeneity between studies due to the greater representation of women in certain age groups and variations in HPV genotypes and testing approaches. We found the distribution of HPV16/18 among all the positive cases to be 27.13%, corresponding to the global pattern [18, 41]. The ability to isolate HPV 16/18, as approached by MOH, is essential in stratifying women at high risk of cervical cancer [42, 43], as these genotypes lead to more than 70% of all cervical cancers [14, 44].

The age group 30 to 39 years had the highest prevalence, which differed from other studies reporting the highest prevalence in the younger age group of less than 25 years [41, 45–47]. It may reflect different lifestyles and sexual activity, with the median age at marriage for Malaysian women being 27 years [48], presumed to be the average age at the beginning of sexual intercourse. Otherwise, the small sample size of those aged between 20–29 years, representing only 1.60% of women attending the screening, may have affected the observed prevalence. Furthermore, women aged 20–29 were found to have the highest likelihood of contracting high-risk HPV infection compared to older women in the multivariate analysis, reflecting findings from various studies [45–47]. However, infection in the younger age group of less than 30 years tends to be transient, and screening in this age group may lead to the detection of lesions that never progress to cancer [49]. Nevertheless, a more vigilant approach to screening women below 40 is needed, as cervical cancer in Malaysia has been reported to peak at age 50 to 65, and many were diagnosed at a later stage [8]. This distribution corresponded with the average progression timeline of about 25 years from persistent infection to cancer [50]. Adhering to the WHO recommendation of two screenings in a woman’s lifetime, first by age 35 and the second by age 45, is considered a high priority [6].

Echoing the prevalence findings, the study showed that residing in urban areas, lower age groups, being Indian or Chinese, and being self-employed were significantly associated with high-risk HPV infection. It declined as age increased, mainly due to the reduction in sexual activity with increasing age and clearance of infection through the immune response [51]. Various studies elsewhere have shown that urban settlers tend to have higher sexual activities, sexual intercourse at an early age, and a higher number of sexual partners compared to rural settlers [52, 53], which may explain the higher likelihood of contracting high-risk HPV infection when compared to rural settling women.

Among all ethnicities, Indian women had the highest prevalence, similar to the finding by Khoo et al. [22]. We found Indians and Chinese to have a significantly higher likelihood of contracting a high-risk HPV infection than Malays. Ethnicity as a significant predictor of HPV infection has been shown in various studies [52–55]. This is in line with the cervical cancer incidence in Malaysia, with the age-standardised incidence rate per 100,000 population being the highest among these two ethnicities [8]. This finding amplifies the need for a targeted screening approach or expanding the public-private partnership to reach more women from these ethnicities, as they were more likely to be underrepresented in the current national screening programme. Further research is warranted to explore the reason behind the high prevalence and likelihood of high-risk HPV infection among non-Malays in Malaysia.

Of important note, the study found that having lower socioeconomic status, represented by education level, income level, and type of occupation (except for being self-employed), was not associated with high-risk HPV infection. This finding was different from the finding by Khoo. et al. [22]. Several studies have proposed that women of lower socioeconomic status often exhibit lower levels of health literacy, including awareness of the significance of health screening and safe sexual practice [54, 56, 57]. However, this observation aligns with other research conducted in Malaysia, where socioeconomic status was generally not identified as a significant predictor of healthcare utilisation. This can be attributed to Malaysia’s robust universal healthcare coverage, which is accessible to a majority of the population [33, 58, 59].

The study utilised the largest sample size from the national public cervical cancer screening registry in estimating the high-risk HPV infection prevalence. It includes data from multiple states and localities in Malaysia. The study has limitations. Those who attended private healthcare facilities and underwent screening were not part of the registry and may have different characteristics. The cross-sectional study design means that the causal relationship between factors and outcomes cannot be established. The crucial risk factors for HPV infection, including individual and partner’s sexual behaviour, were not documented in the registry and could not be investigated in this study. This information, while necessary, is sensitive to be asked for and collected by the registry. This explains the low pseudo r^2^ value found in this study. However, the study aims to identify high-risk groups for HPV infection instead of producing the best predictive model of HPV infection.

The registry can be improved by including HPV vaccination status since the cohort receiving the vaccination has now become susceptible. This is particularly important to evaluate the effectiveness of HPV vaccination, as demonstrated in a recent Malaysian study [60]. In addition, the registry only captured those who agreed to perform the sampling, thus missing out on those who were approached but refused the test. Such information is crucial to understanding those unwilling to participate in an essential public health measure. While the registry allows a comprehensive understanding of HPV infection in Malaysia, the HPV DNA analysis approach means the HPV genotypes were grouped into HPV 16/18 or other high-risk HPV types. Thus, it was impossible to generate prevalence estimates based on individual HPV types and determine the most prevalent types. Finally, a more comprehensive registry would include data on follow-up with women until diagnosis and treatment, which is currently beyond the purview of the national screening registry. Future research should focus on linking the prevalence of HPV infection with cervical cancer incidence to understand Malaysia’s cervical cancer burden better.

## Conclusions

The estimated prevalence of high-risk HPV infection among Malaysian women was 4.53%. About one-third of these infections are attributed to HPV16/18 genotypes, which are known to be oncogenic and highly likely to progress to cervical cancer. This prevalence is similar to the global and neighbouring countries’ rates, with a few high-risk groups identified. Efforts to improve screening uptake in Malaysia should consider a targeted approach to reach these groups. The registry served as a promising platform to provide valuable information in facilitating monitoring and evaluation of the HPV screening progress. It can potentially be expanded to improve this effort and for health policy-making with the ultimate goal of eliminating cervical cancer in Malaysia.

### Supplementary Information

**Additional file 1.** Data collection format of the Malaysian Cervical Cancer Screening Registry.

**Additional file 2.** Univariate and multivariable logistic regression model.

**Additional file 3.** Prevalence of high-risk HPV infection by different genotypes, based on the socio-demographic and economic profile of women attending screening.

**Additional file 4.** Variance inflation factor (VIF) for each independent variable.

## Abbreviations

DNA: Deoxyribonucleic acid
DOSM: Department of Statistics Malaysia
CI: Confidence interval
HPV: Human papillomavirus
MOH: Ministry of Health Malaysia
OR: Odds ratio
Pap: Papanicolaou test
PCR: Polymerase chain reaction
RM: Malaysian Ringgit
WHO: World Health Organization
WPKL: Wilayah Persekutuan Kuala Lumpur
