# Supplementary material for: Prevalence and sociodemographic predictors of high-risk vaginal human papillomavirus infection: findings from a public cervical cancer screening registry
Source: BMC Public Health. 2023 Nov 14;23:2243. doi: 10.1186/s12889-023-17132-2 (PMC10644607; doi:10.1186/s12889-023-17132-2)
Supplement: Supplementary file 1 — Additional file 1. Data collection format of the Malaysian Cervical Cancer Screening Registry. [file 12889_2023_17132_MOESM1_ESM.docx]

Additional file 1. Data collection format of the Malaysian Cervical Cancer Screening Registry.

| Number | Variables | Description |
| --- | --- | --- |
| 1 | Serial number | Client reference number |
| 2 | Clinic name | The name of the primary healthcare clinic where the HPV test is performed. |
| 3 | Registration date | The date of the client’s primary healthcare clinic visit |
| 4 | Date of birth | Client’s date of birth |
| 5 | Age | Client’s age |
| 6 | Nationality | Malaysian or non-Malaysian |
| 7 | Ethnicity | Refers to the client’s ethnicity, such as: |
|  |  | - Malay |
|  |  | - Chinese |
|  |  | - Indian |
|  |  | - Peninsular Natives |
|  |  | - Bajau |
|  |  | - Kadazan Dusun |
|  |  | - Murut |
|  |  | - Other Sabah Natives |
|  |  | - Melanau |
|  |  | - Iban |
|  |  | - Bidayuh |
|  |  | - Other Sarawak Natives |
|  |  | - Other ethnic groups |
| 8 | Education | Refers to the client's highest level of education, such as: |
|  |  | - Never attended school |
|  |  | - Primary |
|  |  | - Secondary |
|  |  | - Certificate |
|  |  | - Diploma |
|  |  | - Degree and above |
| 9 | Occupation | Refers to the client's occupation, such as: |
|  |  | - Government employee |
|  |  | - Private employee |
|  |  | - Self-employed |
|  |  | - Housewife |
|  |  | - Pensioner/retiree |
| 10 | Income | Refers to the gross monthly household income, which is the monthly income of the husband and wife. |
|  |  | - ≤RM 3999 |
|  |  | - RM 4000 – RM 7999 |
|  |  | - ≥RM 8000 |
| 11 | Children | Refers to the current number of children (full-term pregnancy) when undergoing HPV test. |
| 12 | BMI | Refers to the client’s BMI. |
|  |  | Body Mass Index is a measure to assess the client’s body weight in comparison to height [Weight (kg) / Height (m^2^)] |
|  |  | - ˂ 18.5: Underweight |
|  |  | - 18.5 – 24.9: Ideal body weight |
|  |  | - 25 – 30: Overweight |
|  |  | - ≥ 30: Obese |
| 13 | HPV date | The date of HPV test. |
| 14 | Screening type | A new case means undergoing HPV test at a MOH health facility for the first time. |
|  |  | Repeat case means having undergone HPV test at a MOH health facility, including clients with unsatisfactory/negative result that needs to be repeated after 5 years or based on doctor's advice. |
| 15 | Sampling method | Refers to HPV test sampling methods such as: |
|  |  | - Self-sampling |
|  |  | - Assisted by a healthcare provider |
| 16 | Sample delivery point | Refers to laboratories for sample delivery such as: |
|  |  | - IKN-National Cancer Institute |
|  |  | - HKL-Kuala Lumpur Hospital |
|  |  | - MKAK-National Public Health Laboratory |
|  |  | - HSAH-Sultan Abdul Halim Hospital, Sungai Petani |
|  |  | - HRPZ II-Raja Perempuan Zainab II Hospital |
|  |  | - MKAKB-Kota Bharu Public Health Laboratory |
|  |  | - MKAJ-Johor Public Health Laboratory |
|  |  | - Outsource laboratories/Private laboratory |
| 17 | Laboratory sample | Refers to the date when the HPV sample was received by the laboratory designated for sample analysis. |
| 18 | Laboratory results | Refers to the date when the laboratory reports the HPV sample result. |
| 19 | Clinic results | Refers to the date when the primary healthcare clinic receives the HPV result report. |
|  |  | However, for states involved with outsourcing, this date refers to the date when the district health office or divisional health office receives the result. |
| 20 | LTAT | LTAT is the abbreviation for Laboratory Turnaround Time. |
|  |  | Refers to the number of days taken from the date when the sample is received by the laboratory that analysed the sample until the date when the HPV test result is reported. |
| 21 | TTAT | TTAT is the abbreviation for Total Turnaround Time. |
|  |  | Refers to the number of days taken from the date of HPV screening until the date when the primary healthcare clinic receives the HPV test result. |
|  |  | (Note: for outsourced services, TTAT refers to the number of days taken from the date of HPV screening until the date the district health office/divisional health office/area health office receives the HPV test result.) |
| 22 | HPV results | Refers to HPV test results reported by laboratories such as: |
|  |  | - HPV not detected |
|  |  | - Unsatisfactory HPV test |
|  |  | - HPV positive non 16/18 |
|  |  | - HPV positive 16/18 |
| 23 | The date when LBC is performed | Refers to the date when Liquid-based Cytology (LBC) is performed at the primary healthcare clinic. |
| 24 | The date when LBC result is received | Refers to the date when the primary healthcare clinic receives LBC results. |
| 25 | Sample adequacy | Refers to the quality criteria for Liquid-based Cytology (LBC) examination, which is either satisfactory or unsatisfactory based on the presence or absence of endocervical cells. |
|  |  | - Yes-Endocervical cell present |
|  |  | - No-Endocervical cell absent |
| 26 | LBC result (HPV non 16/18 positive) | Refers to the LBC examination for HPV non 16/18 positive conducted by health personnel at the primary healthcare clinic. |
|  |  | The LBC results are as follows: |
|  |  | - Unsatisfactory for evaluation |
|  |  | - NILM |
|  |  | - ASC-US |
|  |  | - LSIL |
|  |  | - ASC-H |
|  |  | - HSIL |
|  |  | - AGC-Atypical glandular cells |
|  |  | - AIS (Adenocarcinoma in-situ) |
|  |  | - SCC (Squamous cell carcinoma) |
|  |  | - Adenocarcinoma |
| 27 | Colposcopy date for HPV non 16/18 positive | Refers to the colposcopy appointment date for clients who are HPV non 16/18 positive. |
| 28 | Colposcopy result for HPV non 16/18 positive | Refers to the result report for HPV non 16/18 positive based on colposcopy conducted at the referral hospital. |
|  |  | - Normal |
|  |  | - Unsatisfactory |
|  |  | - HPV effects/Condylomata Acuminata |
|  |  | - CIN 1 |
|  |  | - CIN 2 |
|  |  | - CIN 3 |
|  |  | - AIS (Adenocarcinoma in-situ) |
|  |  | - Cancer |
| 29 | Colposcopy date for HPV 16/18 positive | Refers to the colposcopy appointment date for clients who are HPV 16/18 positive |
| 30 | Colposcopy result for HPV 16/18 positive | Refers to the result report for HPV 16/18 positive based on colposcopy conducted at the referral hospital. |
|  |  | - Normal |
|  |  | - Unsatisfactory |
|  |  | - HPV effects/Condylomata Acuminata |
|  |  | - CIN 1 |
|  |  | - CIN 2 |
|  |  | - CIN 3 |
|  |  | - AIS (Adenocarcinoma in-situ) |
|  |  | - Cancer |
