# Supplementary material for: Prevalence and sociodemographic predictors of high-risk vaginal human papillomavirus infection: findings from a public cervical cancer screening registry
Source: BMC Public Health. 2023 Nov 14;23:2243. doi: 10.1186/s12889-023-17132-2 (PMC10644607; doi:10.1186/s12889-023-17132-2)
Supplement: Supplementary file 2 — Additional file 2. Univariate and multivariable logistic regression model. [file 12889_2023_17132_MOESM2_ESM.docx]

Additional file 2. Univariate and multivariable logistic regression model.

| **Variables** | | **Univariate** | | | | **Multivariable** | | | |
| --- | --- | --- | --- | --- | --- | --- | --- | --- | --- |
|  |  | **Crude OR** | | **p-value** | | **Adjusted OR** | | **p-value** | |
| **Locality** |  |  |  |  |  |  |  |  |  |
|  | Rural | ref |  |  |  | ref |  |  |  |
|  | Urban |  | 1.90 (1.47-2.45) | <0.001 |  |  | 1.48 (1.09-2.00) | 0.011 | * |
|  |  |  |  |  |  |  |  |  |  |
| **State** |  |  |  |  |  |  |  |  |  |
|  | Johor |  | 2.48 (1.36-4.54) | 0.003 |  |  | 1.72 (0.89-3.30) | 0.104 |  |
|  | Kedah |  | omitted |  |  |  | omitted |  |  |
|  | Kelantan | ref |  |  |  | ref |  |  |  |
|  | Negeri Sembilan |  | 1.36 (0.74-2.52) | 0.325 |  |  | 1.11 (0.58-2.14) | 0.748 |  |
|  | Selangor |  | 0.87 (0.49-1.54) | 0.623 |  |  | 0.54 (0.29-1.02) | 0.058 |  |
|  | WPKL & Putrajaya |  | 2.16 (1.22-3.81) | 0.008 |  |  | 1.31 (0.69-2.48) | 0.415 |  |
|  |  |  |  |  |  |  |  |  |  |
|  |  |  |  |  |  |  |  |  |  |
| **Age group (years)** |  |  |  |  |  |  |  |  |  |
|  | 20-29 |  | 1.30 (0.77-2.20) | 0.330 |  |  | 2.57 (1.48-4.46) | 0.001 | * |
|  | 30-39 |  | 1.12 (0.89-1.41) | 0.341 |  |  | 1.60 (1.24-2.08) | 0.000 | * |
|  | 40-49 |  | 1.49 (1.16-1.92) | 0.002 |  |  | 1.85 (1.42-2.42) | 0.000 | * |
|  | 50-65 | ref |  |  |  | ref |  |  |  |
|  |  |  |  |  |  |  |  |  |  |
| **Ethnicity** |  |  |  |  |  |  |  |  |  |
|  | Malay | ref |  |  |  | ref |  |  |  |
|  | Chinese |  | 1.93 (1.54-2.42) | <0.001 |  |  | 1.67 (1.30-2.13) | 0.000 | * |
|  | Indian |  | 1.87 (1.47-2.39) | <0.001 |  |  | 1.92 (1.49-2.48) | 0.000 | * |
|  | Others |  | 1.76 (1.07-2.87) | 0.025 |  |  | 1.61 (0.98-2.66) | 0.062 |  |
|  |  |  |  |  |  |  |  |  |  |
| **Education level** |  |  |  |  |  |  |  |  |  |
|  | Never attended school/Primary |  | 1.26 (0.88-1.81) | 0.200 |  |  | 1.36 (0.90-2.04) | 0.142 |  |
|  | Secondary |  | 1.19 (1.00-1.43) | 0.050 |  |  | 1.17 (0.94-1.46) | 0.151 |  |
|  | Certificate/Tertiary | ref |  |  |  | ref |  |  |  |
|  |  |  |  |  |  |  |  |  |  |
| **Occupation** |  |  |  |  |  |  |  |  |  |
|  | Self-employed |  | 1.69 (1.22-2.35) | 0.002 |  |  | 1.57 (1.09-2.28) | 0.016 | * |
|  | Government employee | ref |  |  |  | ref |  |  |  |
|  | Private employee |  | 1.39 (1.10-1.75) | 0.005 |  |  | 1.27 (0.98-1.66) | 0.074 |  |
|  | Pensioner/Housewife |  | 1.10 (0.88-1.36) | 0.403 |  |  | 1.09 (0.82-1.43) | 0.557 |  |
|  |  |  |  |  |  |  |  |  |  |
| **Income level** |  |  |  |  |  |  |  |  |  |
|  | <=RM3999 |  | 1.23 (0.89-1.69) | 0.215 |  |  | 1.06 (0.74-1.52) | 0.739 |  |
|  | RM4000-RM7999 |  | 1.23 (0.88-1.72) | 0.234 |  |  | 1.18 (0.84-1.67) | 0.343 |  |
|  | >=RM8000 | ref |  |  |  | ref |  |  |  |

Note. OR = Odds ratio, 95% CI = 95% confidence interval, RM = Malaysian Ringgit, WPKL = Wilayah Persekutuan Kuala Lumpur.
* p-value < 0.05
# Multicollinearity with locality
