# Supplementary material for: Prevalence and sociodemographic predictors of high-risk vaginal human papillomavirus infection: findings from a public cervical cancer screening registry
Source: BMC Public Health. 2023 Nov 14;23:2243. doi: 10.1186/s12889-023-17132-2 (PMC10644607; doi:10.1186/s12889-023-17132-2)
Supplement: Supplementary file 3 — Additional file 3. Prevalence of high-risk HPV infection by different genotypes, based on the socio-demographic and economic profile of women attending screening. [file 12889_2023_17132_MOESM3_ESM.docx]

Additional file 3. Prevalence of high-risk HPV infection by different genotypes, based on the socio-demographic and economic profile of women attending screening.

| **Variables** | | **Total, n** | **High-risk HPV not detected** | | **HPV Positive 16/18** | | **High-risk non-16/18 HPV Positive** | | **Unsatisfactory HPV test** | | **Missing** | |
| --- | --- | --- | --- | --- | --- | --- | --- | --- | --- | --- | --- | --- |
|  |  |  | **n** | **%** | **n** | **%** | **n** | **%** | **n** | **%** | **n** | **%** |
| **Total** | Overall | 36,738 | 34,005 | 92.56 | 452 | 1.23 | 1,214 | 3.30 | 911 | 2.48 | 156 | 0.42 |
| **Locality†** |  |  |  |  |  |  |  |  |  |  |  |  |
|  | Rural | 3,489 | 3,295 | 94.44 | 23 | 0.66 | 65 | 1.86 | 84 | 2.41 | 22 | 0.63 |
|  | Urban | 23,248 | 21,307 | 91.65 | 349 | 1.50 | 947 | 4.07 | 562 | 2.42 | 83 | 0.36 |
|  | Unrecorded^#^ | 10,001 | 9,403 | 94.02 | 80 | 0.80 | 202 | 2.02 | 265 | 2.65 | 51 | 0.51 |
| **State** |  |  |  |  |  |  |  |  |  |  |  |  |
|  | Johor | 979 | 854 | 87.23 | 11 | 1.12 | 63 | 6.44 | 26 | 2.66 | 25 | 2.55 |
|  | Kedah | 9,392 | 8,829 | 94.01 | 74 | 0.79 | 192 | 2.04 | 248 | 2.64 | 49 | 0.52 |
|  | Kelantan | 2,410 | 2,275 | 94.40 | 13 | 0.54 | 37 | 1.54 | 77 | 3.20 | 8 | 0.33 |
|  | Negeri Sembilan | 1,252 | 1,149 | 91.77 | 20 | 1.60 | 35 | 2.80 | 39 | 3.12 | 9 | 0.72 |
|  | Selangor | 4,997 | 4,794 | 95.94 | 41 | 0.82 | 106 | 2.12 | 39 | 0.78 | 17 | 0.34 |
|  | WPKL & Putrajaya | 17,708 | 16,104 | 90.94 | 293 | 1.65 | 781 | 4.41 | 482 | 2.72 | 48 | 0.27 |
| **Age group (years)** |  |  |  |  |  |  |  |  |  |  |  |  |
|  | 20-29 | 587 | 529 | 90.12 | 14 | 2.39 | 11 | 1.87 | 22 | 3.75 | 11 | 1.87 |
|  | 30-39 | 21,478 | 19,901 | 92.66 | 283 | 1.32 | 713 | 3.32 | 490 | 2.28 | 91 | 0.42 |
|  | 40-49 | 11,073 | 10,194 | 92.06 | 122 | 1.10 | 389 | 3.51 | 323 | 2.92 | 45 | 0.41 |
|  | 50-65 | 3,600 | 3,381 | 93.92 | 33 | 0.92 | 101 | 2.81 | 76 | 2.11 | 9 | 0.25 |
| **Ethnicity** |  |  |  |  |  |  |  |  |  |  |  |  |
|  | Malay | 30,638 | 28,564 | 93.23 | 340 | 1.11 | 842 | 2.75 | 759 | 2.48 | 133 | 0.43 |
|  | Chinese | 3,046 | 2,764 | 90.74 | 46 | 1.51 | 177 | 5.81 | 47 | 1.54 | 12 | 0.39 |
|  | Indian | 2,447 | 2,145 | 87.66 | 51 | 2.08 | 159 | 6.50 | 84 | 3.43 | 8 | 0.33 |
|  | Others | 591 | 519 | 87.82 | 14 | 2.37 | 36 | 6.09 | 19 | 3.21 | 3 | 0.51 |
|  | Missing | 16 | 13 | 81.25 | 1 | 6.25 | 0 | 0 | 2 | 12.50 | 0 | 0 |
| **Education level†** |  |  |  |  |  |  |  |  |  |  |  |  |
|  | Never attended school/Primary | 655 | 607 | 92.67 | 5 | 0.76 | 32 | 4.89 | 9 | 1.37 | 2 | 0.31 |
|  | Secondary | 4,816 | 4,467 | 92.75 | 71 | 1.47 | 181 | 3.76 | 75 | 1.56 | 22 | 0.46 |
|  | Certificate/Tertiary | 5,923 | 5,526 | 93.30 | 66 | 1.11 | 194 | 3.28 | 93 | 1.57 | 44 | 0.74 |
|  | Unrecorded^#^ | 25,344 | 23,405 | 92.35 | 310 | 1.22 | 807 | 3.18 | 734 | 2.90 | 88 | 0.35 |
| **Occupation†** |  |  |  |  |  |  |  |  |  |  |  |  |
|  | Self-employed | 758 | 690 | 91.03 | 13 | 1.72 | 38 | 5.01 | 11 | 1.45 | 6 | 0.79 |
|  | Government employee | 3,675 | 3,433 | 93.41 | 38 | 1.03 | 114 | 3.10 | 71 | 1.93 | 19 | 0.52 |
|  | Private employee | 2,665 | 2,461 | 92.35 | 38 | 1.43 | 113 | 4.24 | 33 | 1.24 | 20 | 0.75 |
|  | Pensioner/Housewife | 4,294 | 4,014 | 93.48 | 53 | 1.23 | 142 | 3.31 | 62 | 1.44 | 23 | 0.54 |
|  | Unrecorded^#^ | 25,346 | 23,407 | 92.35 | 310 | 1.22 | 807 | 3.18 | 734 | 2.90 | 88 | 0.35 |
| **Income level†** |  |  |  |  |  |  |  |  |  |  |  |  |
|  | <=RM3999 | 6,656 | 6,189 | 92.98 | 91 | 1.37 | 236 | 3.55 | 95 | 1.43 | 45 | 0.68 |
|  | RM4000-RM7999 | 3,637 | 3,375 | 92.80 | 40 | 1.10 | 137 | 3.77 | 69 | 1.90 | 16 | 0.44 |
|  | >=RM8000 | 1,091 | 1,027 | 94.13 | 11 | 1.01 | 33 | 3.02 | 13 | 1.19 | 7 | 0.64 |
|  | Unrecorded^#^ | 25,354 | 23,414 | 92.35 | 310 | 1.22 | 808 | 3.19 | 734 | 2.90 | 88 | 0.35 |
| **Sampling method** |  |  |  |  |  |  |  |  |  |  |  |  |
|  | Assisted by HCP | 826 | 755 | 91.40 | 8 | 0.97 | 33 | 4.00 | 23 | 2.78 | 7 | 0.85 |
|  | Self-sampling | 35,872 | 33,215 | 92.59 | 443 | 1.23 | 1,180 | 3.29 | 885 | 2.47 | 149 | 0.42 |
|  | Missing | 40 | 35 | 87.50 | 1 | 2.50 | 1 | 2.50 | 3 | 7.50 | 0 | 0 |
| **Year of HPV screening** |  |  |  |  |  |  |  |  |  |  |  |  |
|  | 2019 | 17,493 | 16,205 | 92.64 | 229 | 1.31 | 562 | 3.21 | 451 | 2.58 | 46 | 0.26 |
|  | 2020 | 9,073 | 8,299 | 91.47 | 97 | 1.07 | 290 | 3.20 | 336 | 3.70 | 51 | 0.56 |
|  | 2021 | 10,172 | 9,501 | 93.40 | 126 | 1.24 | 362 | 3.56 | 124 | 1.22 | 59 | 0.58 |
| **Number of children** |  |  |  |  |  |  |  |  |  |  |  |  |
|  | 0 | 2,444 | 2,128 | 87.07 | 54 | 2.21 | 171 | 7.00 | 82 | 3.36 | 9 | 0.37 |
|  | 1 | 4,813 | 4,407 | 91.56 | 72 | 1.50 | 189 | 3.93 | 119 | 2.47 | 26 | 0.54 |
|  | 2 | 8,820 | 8,176 | 92.70 | 109 | 1.24 | 273 | 3.10 | 220 | 2.49 | 42 | 0.48 |
|  | 3 | 9,816 | 9,142 | 93.13 | 120 | 1.22 | 297 | 3.03 | 213 | 2.17 | 44 | 0.45 |
|  | 4 | 6,246 | 5,855 | 93.74 | 58 | 0.93 | 157 | 2.51 | 150 | 2.40 | 26 | 0.42 |
|  | >4 | 4,380 | 4,095 | 93.49 | 36 | 0.82 | 123 | 2.81 | 118 | 2.69 | 8 | 0.18 |
|  | Missing | 219 | 202 | 92.24 | 3 | 1.37 | 4 | 1.83 | 9 | 4.11 | 1 | 0.46 |
| Note. † = Information on locality, education level, income level and occupation was only collected from August 2020 onwards, rendering many unrecorded cases marked as ^#^. HCP = healthcare provider, RM = Malaysian Ringgit, WPKL = Wilayah Persekutuan Kuala Lumpur, n = number, % = percentage. | | | | | | | | | | | | |
