# Supplementary material for: Prevalence and sociodemographic predictors of high-risk vaginal human papillomavirus infection: findings from a public cervical cancer screening registry
Source: BMC Public Health. 2023 Nov 14;23:2243. doi: 10.1186/s12889-023-17132-2 (PMC10644607; doi:10.1186/s12889-023-17132-2)
Supplement: Supplementary file 4 — Additional file 4. Variance inflation factor (VIF) for each independent variable. [file 12889_2023_17132_MOESM4_ESM.docx]

Additional file 4. Variance inflation factor (VIF) for each independent variable.

| **Variable** | **VIF** | **SQRT VIF** | **Tolerance** | **R-Squared** |
| --- | --- | --- | --- | --- |
| Strata | 1.12 | 1.06 | 0.8892 | 0.1108 |
| State | 1.10 | 1.05 | 0.9106 | 0.0894 |
| Age group | 1.16 | 1.08 | 0.8594 | 0.1406 |
| Ethnicity | 1.07 | 1.04 | 0.9320 | 0.0680 |
| Education level | 1.47 | 1.21 | 0.6798 | 0.3202 |
| Occupation | 1.22 | 1.10 | 0.8205 | 0.1795 |
| Income level | 1.33 | 1.15 | 0.7530 | 0.2470 |
| Mean VIF | 1.21 |  |  |  |
| Note. VIF = Variance inflation factor, SQRT = Squared root. | | | | |
